# Pharmacokinetics and Main Metabolites of Anwulignan in Mice

**DOI:** 10.3389/fphar.2022.929177

**Published:** 2022-07-05

**Authors:** Cong Chen, Yanbo Feng, Han Li, Hao Lin, Shu Jing, He Li, Chunmei Wang, Jianguang Chen, Jinghui Sun

**Affiliations:** College of Pharmacy, Beihua University, Jilin, China

**Keywords:** *Schisandra sphenanthera*, anwulignan, pharmacokinetics, metabolites, UHPLC-Q-Orbitrap-MS

## Abstract

Anwulignan is a representative component of Chinese traditional medicine *Schisandra sphenanthera*, with strong pharmacological activities. However, there are few reports on its pharmacokinetics and metabolites in the body. In this study, a metabolomic method based on UHPLC-Q-Orbitrap-MS was used to study the pharmacokinetics of anwulignan in the blood, organs, urine, and feces samples of mice after the intragastric administration of anwulignan (10 mg/kg). The pharmacokinetic parameters were calculated, and the distribution characteristics and main metabolites of anwulignan in the body of mice were analyzed. The results showed that the retention time of anwulignan in the body of mice was longer (*t*_1/2_ = 7.1 h), and anwulignan was widely distributed in the body (V_z/F_ = 32.81 L/kg), especially in the liver. The order of anwulignan concentration in the tissues of mice from high to low was the liver > heart > brain > kidney > lung > spleen. Anwulignan was mainly excreted through the digestive tract in the form of its prototype and metabolites, indicating that it might experience an enterohepatic circulation. A total of seven metabolites were identified, and the demethylation, hydroxylation, dehydroxylation, and demethoxylation were considered to be the main metabolic ways of anwulignan in the body of mice.

## Graphical Abstract

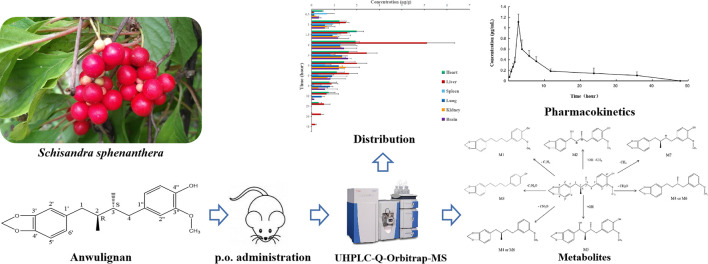

## Introduction

*Schisandra sphenanthera*, the mature and dry fruit of *S. sphenanthera* Rehd. Et Wils, has been used as a representative tonic drug in China for 2,000 years (Jiang et al., 2019). The active components of *S. sphenanthera* include lignans, volatile oil, organic acids, and polysaccharides, among which lignans are the main active components, with some pharmacological effects, such as bacteriostasis, sedation and hypnosis, anti-aging, anti-oxidation, and hepatoprotection (Jiang et al., 2016; Zhang et al., 2021). Wuzhi Tablets, an alcohol-extracted preparation of *S. sphenanthera*, is used for the treatment of hepatitis and liver insufficiency caused by viruses and drugs in clinic (Chen et al., 2019). Anwulignan is a representative lignan component of *S. sphenanthera* and a characteristic component for the identification of *S. sphenanthera*, and also one of the main components in Wuzhi Tablets (Lu and Liu, 1992; Yang et al., 2014). However, there are few reports on the efficacy of anwulignan. It was found in our previous study that anwulignan, with a good antioxidant effect, could significantly alleviate the liver injury induced by intestinal ischemia reperfusion in rats (Lin et al., 2020), regulate the immune function of aging mice induced by d-galactose by activating Nrf2/ARE pathway and regulating caspase-3, BCL2, and Bax (Li et al., 2020), and alleviate the heart injury induced by oxidative stress (Liu et al., 2020a), with an anti-fatigue effect (Liu et al., 2020b; Zhang et al., 2020), demonstrating that it is worthy of further development and utilization. The pharmacokinetic study of anwulignan is particularly important to clarify the pharmacological mechanism of anwulignan *in vivo*, and not only the pharmacological effect of anwulignan on the human body can be observed but also more appropriate administration dosage and method can be found according to the absorption, distribution, and excretion law of anwulignan in various tissues and organs in the study so as to achieve the best curative effect. Therefore, it has a broad prospect for clinical research and social application to explore the action mechanism and potential targets of anwulignan based on pharmacokinetics. However, there are few studies on the pharmacokinetics of anwulignan, especially its biotransformation process *in vivo*.

The analysis of anwulignan is mainly carried out by HPLC and UV methods, and both of them are used for the study on the determination of its *in vitro* content. The main purpose of ultra-high-performance liquid chromatography–quadrupole orbitrap mass spectrometry (UHPLC-Q-Orbitrap-MS) analysis is to provide a quantitative method for candidate drugs, so as to obtain accurate and reliable conclusions in the study of pharmacokinetics. In this study, UHPLC-Q-Orbitrap-MS was used to analyze the pharmacokinetic characteristics, the distribution in tissues, and the main metabolites of anwulignan in the body of mice to seek the potentially active metabolites of anwulignan *in vivo*, which was expected to provide support in data and a reference for the pharmacodynamic material basis of anwulignan as a candidate drug.

## Materials and Methods

### Chemicals and Reagents

The materials used in this study were as follows: anwulignan and schisantherin A (>98.0%, Chengdu Preferred Biotechnology Limited Company, Chengdu, Sichuan, China); methanol and ethyl acetate (HPLC–grade, TEDIA, Cincinnati, OH, United States); and de-ionized water (prepared by a Milli-Q Water purification system, Millipore, MA, United States).

### Preparation of Plasma and Tissue Standards

Male ICR mice (18–22 g, Chang Chun Yisi Experimental Animal Technology Co., Ltd.) were used for experiments after the isolation and adaptation in our laboratory for 1 week. The mice were raised under the standard laboratory conditions for 1 week before experiments, with a free access to water and food. All experimental procedures were approved by Beihua University Laboratory Animal Ethics Committee (No. 20190907) and performed in accordance with the Guide for the Care and Use of Laboratory Animals.

Using methanol as the solvent, the concentration of anwulignan standard and schisandrin A (internal standard, IS) was 100 and 4 μg/ml, respectively. The different volumes of anwulignan standard stock solution and 200 μl schisandrin A were added into 200 μl of blank plasma of mice (or heart, liver, spleen, lung, kidney, and brain tissue homogenate, and a normal saline homogenate at the ratio of 1:9, w/v), which were mixed evenly. The solutions were centrifuged at 3,000 rpm for 10 min, then added with double volumes of methanol and swirled for 30 s, and centrifuged at 13,000 rpm for 10 min for obtaining the supernatants. The supernatants were blown dry until no liquid in them was found. The dried supernatant sentiments were dissolved in methanol up to a volume of 2 ml, respectively, and the solutions were swirled for 30 s and then filtered through a filter with a diameter of 0.45 μm into 1.5-ml sample bottles. Finally, the plasma (tissue) standard solutions at the final concentrations of anwulignan of 0.01, 0.02, 0.05, 0.1, 0.2, 0.5, 1, 2, and 5 μg/ml were prepared, respectively, and the concentration of schisandrin A in each sample was 4 μg/ml. A volume of 10 μl of each sample was analyzed by LC/MS to obtain the calibration curve (repeated three times). The 0.05, 0.5, and 5 μg/ml plasma (tissue) standard solutions were selected as the quality control (QC) samples for the method validation in this study.

### Administration of Anwulignan and Collection and Preparation of Plasma and Organ Samples

Carboxymethylcellulose sodium (CMC-Na, 0.5 g) was dissolved in 1,000 ml distilled water, and 10 mg anwulignan was dissolved in 10 ml of the CMC-Na solution prepared above, at the concentration of 1 mg/ml. Sixty mice were fasted for 12 h, and then intragastrically administered with 10 mg/kg anwulignan. At 0.5, 1, 1.5, 2, 3, 4, 6, 8, 12, 24, 36, and 48 h after the administration of anwulignan, five mice were randomly selected at each time point for the preparation of homogenates. The selected mice were placed in a desiccator in which 10 ml ether was placed at the lower part. After the mice were anesthetized (about 0.5 min), the blood samples of mice were collected by removing eyeballs, and then their heart, liver, spleen, lung, kidney, and brain tissues were taken. The blood plasma and tissue homogenates were prepared according to the method in the preparation of plasma and tissue standards, and 200 μl of schisandrin A (4 μg/ml) was added into each of the plasma and tissue homogenates for the preparation of the samples to be tested.

### Collection and Preparation of Urine and Feces Samples of Mice After the Intragastric Administration

Five mice were selected and intragastrically administered with anwulignan (10 mg/kg), and then the mice were placed in a mouse metabolism cage and fasted, but with a free access to water. The urine and feces were collected during 0–2 h, 2–4 h, 4–8 h, 8–12 h, 12–24 h, and 24–48 h after the administration of anwulignan, respectively. The urine samples were blown dry, and the concentrated urine was extracted three times with an appropriate amount of ethyl acetate. The five extracts were mixed and blown dry with nitrogen, then the dried extract was added with 1 ml methanol and dissolved by vortexing. The extract-methanol solution was passed through a 0.45 μm microporous membrane and the filtrate was collected, and the volume of filtrate was adjusted to 2 ml with methanol for testing. Each of the collected feces samples was added with 5 ml of water and ground, and then added with 10 ml of ethyl acetate and extracted three times. The three extracts were mixed. The mixed extract was blown dry with nitrogen, and then the dried extract was dissolved in a small amount of methanol. The extract-methanol solution was filtered through a 0.45 μm microporous membrane, and the volume of the collected filtrate was adjusted to 2 ml with methanol for testing.

### UHPLC-Q-Orbitrap-MS/MS Conditions

An ultimate 3,000 ultra-high-performance liquid chromatography system (Thermo, San Jose, CA, United States) was used for the separation by liquid chromatography. The separation column of HPLC was C18 column (100 mm × 30 mm, 18 μm), and the column temperature was set at 32°C and the absorbance at 230 nm. Methanol and water were used as mobile phase A and B, respectively. The gradient elution procedures were set as follows: 0–15 min (75%–90% A), 10–15 min (90%–100% A), and 15–18 min (100%–75% A). The flow rate was set at 1 ml/min and the injection volume was 10 μl. The UHPLC system was coupled to a Q-Orbitrap-MS/MS mass spectrometer.

Q-Orbitrap-MS/MS (Thermo, San Jose, CA, United States) was used for the mass spectrometry in positive-ion mode. The capillary voltage was set at +4.0 kV and the capillary temperature at 350°C. The parameters of the ion source were set as follows: the sheath gas flow was 40 Arb, the auxiliary gas flow 10 Arb, and the purge gas flow 1 Arb. A single-ion monitor (SIM) mode was used for the analysis of anwulignan and IS, and the full-scan MS data were used for the analysis of metabolites by setting the *m/z* as 150–2,000 Da, the resolution as 70,000, automatic gain control (AGC), the target value as 1 × 10^6^, and the maximum injection time (IT) as 100 ms. Full-MS/ddMS^2^ mode was set to get MS/MS data, in which the resolution was 17,500, the AGC target was 1 × 10^5^, the maximum IT was 50 ms, and the normalized collision energy (NCE) was 25–45.

### Multivariate Data Analysis and Metabolite Identification

LC-MS data were extracted, filtered, and normalized using Thermo software Xcalibur (version 4.3) to obtain the molecular weight, retention time, and absorption peak area of the compounds in each sample. SIMCA 14.1 software (Umetrics, Kinnelon, NJ, United States) was used for the analysis of multivariate data, and principal component analysis (PCA) and orthogonal partial least squares discrimination analysis (OPLS-DA) were used to look for the differential compounds among the samples by analyzing the score charts and scatter plots. Xcalibur was used to compare the molecular weight, retention time, and molecular characterization of fragmented ions of the differential compounds by MS/MS mass spectrometry for their identification and structure elucidation.

### Specificity

Six different batches of mouse blank plasma (tissues), and the blank plasma and tissue homogenates with anwulignan and IS were compared for the specificity investigation, in which whether the plasma could interfere with the retention time and peak area of anwulignan and IS was observed.

### Standard Curve and Limit of Quantitation

The plasma (tissue) standard solution of anwulignan in each concentration gradient was selected, and the standard curve was obtained by plotting the relation curve between the peak area of anwulignan/IS and the concentration of anwulignan. The peak area of anwulignan was recorded, and the signal-to-noise ratio (SNR) 10:1 was used as the LOQ.

### Precision and Accuracy

The precision and accuracy were evaluated by the intra-day analysis (three different concentrations of QC samples, six replicates for each concentration, and determined three times) and the inter-day analysis (consecutively for 3 days). The relative error (RE%) calculated based on the peak area should be less than 20%.

### Matrix Effect and Stability

Three quality control samples at the different concentrations were detected by HPLC method, in which six parallel samples were set for each concentration, to obtain the peak area set 1. Thirty microliters of the above standard solution at three concentrations were added to 150 μl of the extracted blank mouse plasma and then 100 μl water was added to the standard plasma solution, well mixed by a vortex mixer, in which six parallel samples were set for each concentration, and the solution was detected to obtain the peak area set 2. The matrix effect was calculated according to the value of set 2/set 1. The stability of anwulignan was evaluated by analyzing six duplicate samples from each of the three quality control samples at the different concentrations under the different conditions, including placing at room temperature for 4 h, freezing at 80°C for 30 days, and three freeze–thaw cycles (from −20°C to room temperature), to record the relative peak area of each color spectrum peak for the calculation of its stability.

### Recovery

The sample addition recovery method was used for the recovery test. Three quality control samples at the different concentrations (five samples for each concentration) were taken and anwulignan was added to the plasma (tissues), and then their chromatogram peak areas were measured under the UHPLC-Q-Orbitrap-MS/MS conditions. The measured peak areas were compared with those of the blank plasma (tissues) after the treatment with the same amount of anwulignan for the calculation of the recovery rate.

## Results

### Method Validation

**FIGURE 1:**
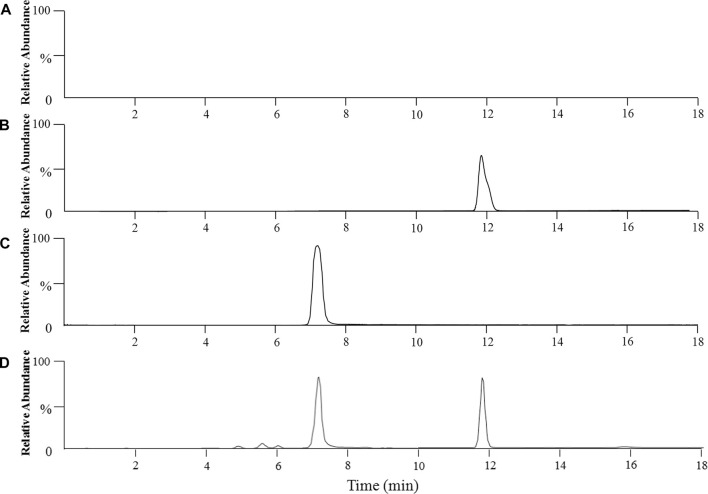
Representative chromatograms of anwulignan and schisandrin A in the plasma of mice. Note: **(A)** blank plasma; **(B)** blank plasma spiked with anwulignan; **(C)** blank plasma spiked with schisandrin A; **(D)** a mice plasma sample collected after administration of a single dosage of anwulignan (3 h).

Figure 1 shows the extracted ion chromatograms (EIC) of anwulignan (*m/z* 329) and schisantherin A (*m/z* 415). The results showed that their retention time was 11.91 and 7.42 min for them, respectively, and no endogenous interference was found within the retention time of anwulignan and IS.

**FIGURE 2:**
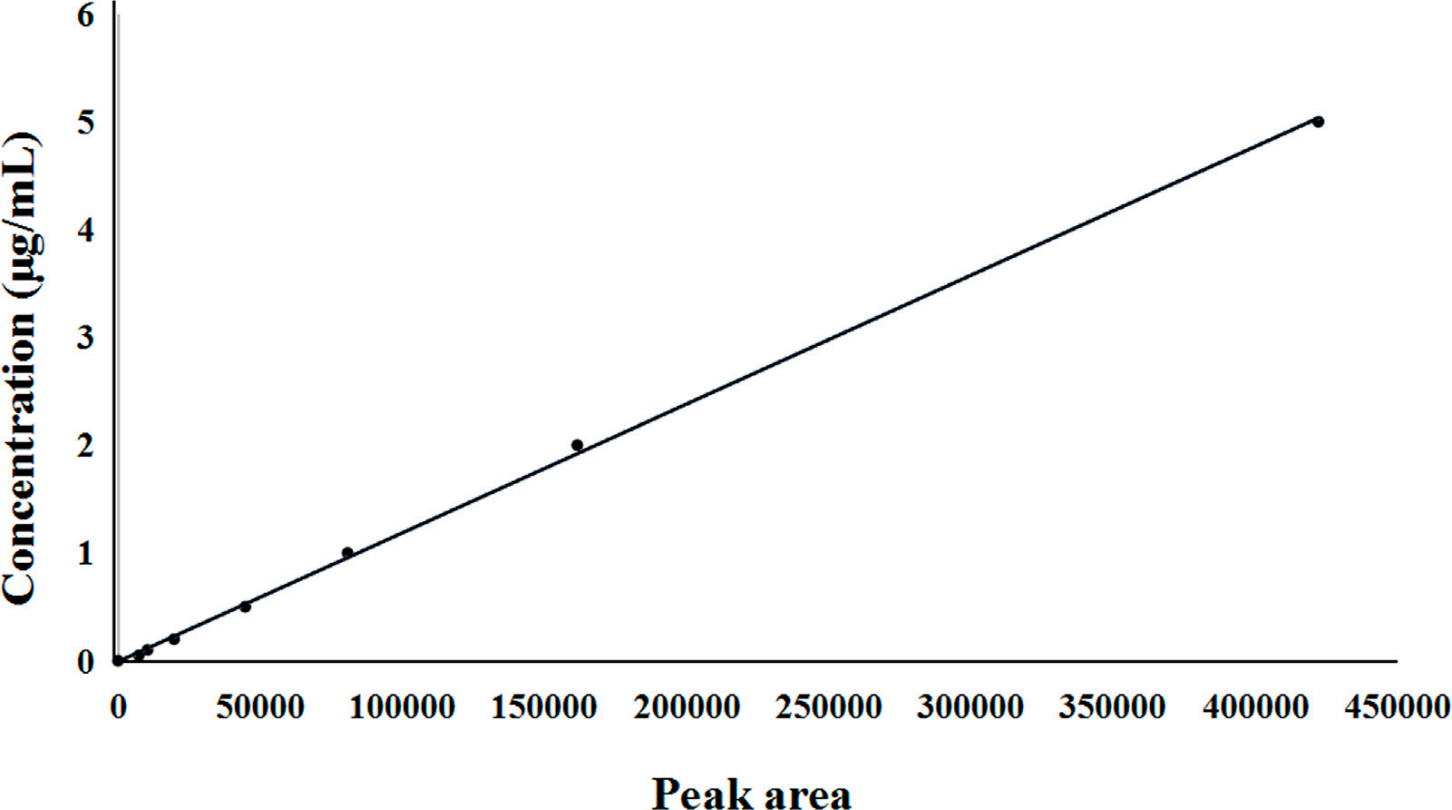
Standard curve of anwulignan in plasma.

As shown in Table 1, the linear range of anwulignan in the plasma (*n* = 3) was 0.01–5 μg/ml, and that of anwulignan in the heart, liver, kidney, spleen, and brain homogenates (*n* = 3) was 0.02–5.0 μg/ml; under the selected chromatographic conditions, the LOQ was 0.02 μg/ml. The plasma standard curve of anwulignan is shown in Figure 2.

**TABLE 1:** Regression equation of anwulignan in plasma and tissue samples.

Sample	Regression equation	R^2^	Linear range (µg/ml)
Plasma	Y = 0.00005X−0.0492	0.9994	0.01–5.00
Heart	Y = 0.00006X + 0.0387	0.9991	0.02–5.00
Liver	Y = 0.00010X + 0.0154	0.9994	0.02–5.00
Lung	Y = 0.00004X + 0.0154	0.9993	0.02–5.00
Kidney	Y = 0.00004X + 0.0568	0.9992	0.02–5.00
Spleen	Y = 0.00004X−0.0372	0.9992	0.02–5.00
Brain	Y = 0.00005X + 0.0267	0.9993	0.02–5.00

The matrix effect of anwulignan sample was 94.6%–98.7%, and the results of its short-term and long-term stability investigation are shown in Table 2. As shown in Table 3, the RSD value for evaluating the precision of anwulignan in each sample was less than 5.6%, the RSD value for the accuracy was less than 6.2%, the average recovery was between 92.7% and 98.5%, and the stability was less than 3.4%, indicating that the results obtained by the method should be reliable.

**TABLE 2:** Matrix effect and stability of anwulignan in plasma and tissue samples.

Sample	QC (µg/ml)	Matrix effect (%)	Stability (%)		
			Short term	Long term	3 Freeze–thaw cycles
Plasma	0.05	95.7	95.6	95.4	97.7
	0.5	96.5	98.5	91.6	98.5
	5	94.7	88.5	87.6	99.4
Heart	0.05	97.6	94.5	93.5	99.2
	0.5	96.7	92.3	96.2	97.5
	5	95.9	96.7	97.4	98.8
Liver	0.05	97.2	94.5	89.5	99.2
	0.5	94.8	95.6	96.7	96.6
	5	98.2	91.5	98.2	97.5
Kidney	0.05	94.6	89.5	98.6	97.9
	0.5	97.9	96.7	89.6	97.7
	5	97.5	93.2	92.5	99.5
Spleen	0.05	98.7	94.5	93.4	99.2
	0.5	96.4	87.5	95.8	98.1
	5	96.5	95.6	92.7	98.5
Brain	0.05	98.1	94.2	95.8	97.4
	0.5	97.3	97.2	94.6	99.6
	5	97.6	93.5	93.5	97.5
Lung	0.05	95.7	87.5	92.3	98.6
	0.5	97.2	92.3	95.4	97.8
	5	96.5	88.6	93.2	97.3

**TABLE 3:** Precision, accuracy, and recover of the method used for the determination of anwulignan.

Sample	QC (µg/ml)	Precision (%)		Accuracy (%)	Recovery (%)
		Inter-day	Intra-day		
Plasma	0.05	3.6	5.0	5.3	92.7
	0.5	−1.2	3.0	4.6	97.0
	5	2.1	4.4	1.2	95.6
Heart	0.05	3.4	−4.7	−3.2	98.4
	0.5	2.3	−2.8	−2.7	94.3
	5	1.3	3.7	2.9	95.2
Liver	0.05	−4.4	5.4	4.8	98.5
	0.5	2.6	−4.3	−3.2	98.2
	5	−3.3	−3.2	−4.5	97.3
Kidney	0.05	5.1	5.6	−4.7	96.3
	0.5	2.7	3.2	3.9	95.3
	5	−3.8	−4.2	6.4	97.2
Spleen	0.05	−2.1	4.9	1.9	98.3
	0.5	−1.6	−5.5	−3.8	98.5
	5	−2.4	4.2	5.6	97.6
Brain	0.05	4.2	5.6	3.7	94.5
	0.5	−3.3	−1.2	−5.1	96.2
	5	2.8	4.5	4.8	98.2
	0.05	2.1	3.2	5.2	93.5
Lung	0.5	3.6	5.2	−3.8	97.6
	5	4.7	1.2	−2.7	96.8

### Pharmacokinetics and Distribution of Anwulignan *In Vivo*

**FIGURE 3:**
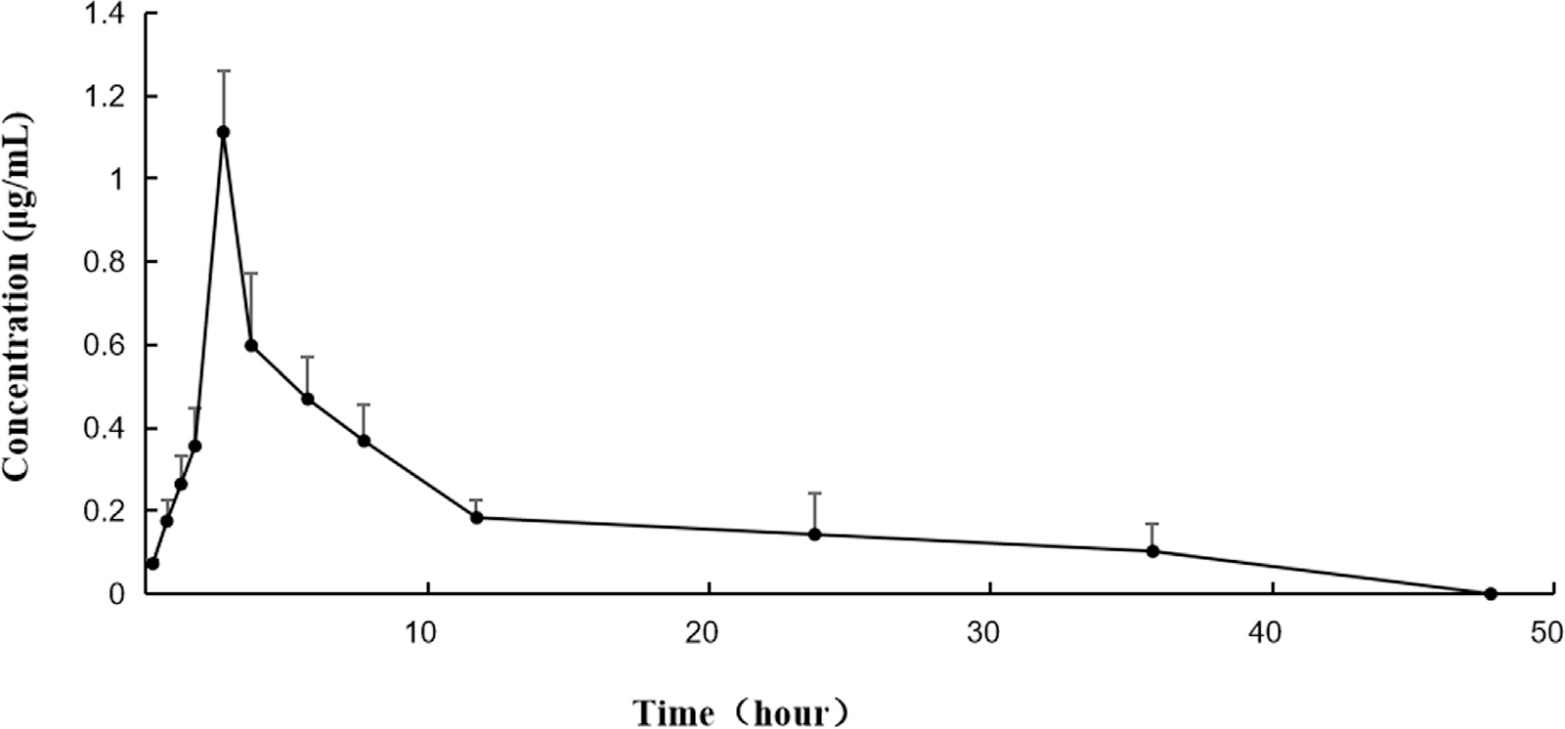
Concentration–time curve of anwulignan in mice after the intragastric administration of anwulignan.

**FIGURE 4:**
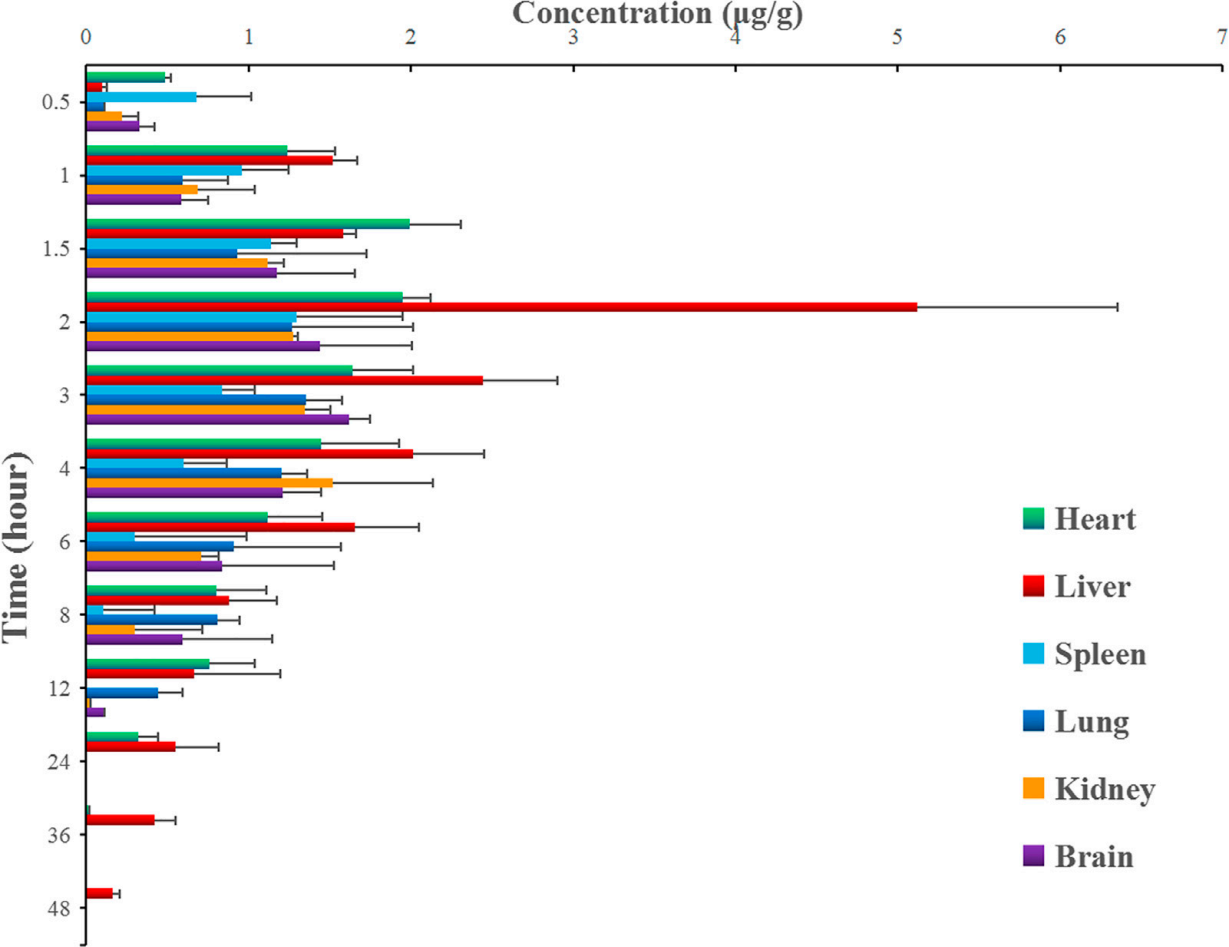
Concentrations of anwulignan in the different tissues of mice after the intragastric administration of anwulignan.

After the intragastric administration of anwulignan, the peak area of each time period detected by HPLC was substituted into the standard curve to obtain the corresponding concentrations of anwulignan. The data obtained were analyzed with pharmacokinetics software DAS (version 3.2.8), and the results are shown in Figure 3, the pharmacokinetic parameters are shown in Table 4, and the contents of anwulignan in the different tissues are shown in Figure 4. As shown in Figure 3, the plasma concentration of anwulignan was the highest at 3 h after the intragastric administration of anwulignan, and lasted for a long time. Its Tmax and T_1/2_ calculated by the judgment method of non-compartment model were 3.00 and 7.10 h, respectively, indicating that the duration of anwulignan is long in the body of mice. The apparent distribution volume of anwulignan was 32.81 ± 4.79 L/kg, indicating that anwulignan has a strong lipophilicity, so it is easy for it to enter cells and be widely distributed in tissues and organs rich in fat. Its AUC_0−t_ and AUC_0−∞_ were 9.37 ± 2.36 and 12.58 ± 3.51 mg/Lh, respectively, indicating that anwulignan may enter the systemic circulation more and be absorbed more completely. Its CL_Z/F_ was 0.80 ± 0.23 L/h/kg, indicating that the clearance rate of anwulignan in the body is slow. The relative bioavailability of anwulignan was 23.8%, indicating that it may be metabolized through an obvious hepatointestinal circulation. As shown in Figure 4, anwulignan could be detected in the heart, liver, spleen, lung, kidney, and brain tissues of mice, and the order of the total concentration of anwulignan from high to low within 48 h was liver > heart > brain > kidney > lung > spleen (Table 5). At 2 h, the concentration of anwulignan in the liver was the highest, and the content of anwulignan in the liver is higher than that in the other tissues. The content in the liver and heart tissues was the highest within 2 h, and that in the liver, heart, and brain tissues was higher at 3 h.

**TABLE 4:** Primary pharmacokinetic parameters of anwulignan in mice after the intragastric administration of anwulignan.

Parameter	Value
C_max_ (mg/L)	1.11 ± 0.20
T_max_ (h)	3.00 ± 0.15
t_1/2_ (h)	7.10 ± 1.89
AUC_0-t_ (mg/L·h)	9.37 ± 2.36
AUC_0-∞_ (mg/L·h)	12.58 ± 3.51
MRT_0-t_ (h)	13.65 ± 4.62
MRT_0-∞_ (h)	32.90 ± 6.35
V_z/F_ (L/kg)	32.81 ± 4.79
CL_z/F_ (L/h/kg)	0.80 ± 0.23

**TABLE 5:** Concentrations of anwulignan in the different tissues of mice after the intragastric administration of anwulignan.

Time	Heart	Liver	Spleen	Lung	Kidney	Brain
0.5 h	0.4865	0.0954	0.6794	0.1025	0.2214	0.3244
1 h	1.2377	1.5179	0.9607	0.5906	0.6872	0.5852
1.5 h	1.9920	1.5793	1.1341	0.9274	1.1125	1.1745
2 h	1.9513	5.1179	1.2974	1.2680	1.3420	1.4400
3 h	1.6376	2.4461	0.8331	1.3544	1.5175	1.6175
4 h	1.4442	2.0100	0.6012	1.2006	0.7064	1.2109
6 h	1.1159	1.6523	0.3012	0.9106	0.3012	0.8329
8 h	0.8021	0.8794	0.1025	0.8080	0.0160	0.5890
12 h	0.7590	0.6657	—	0.4384	—	0.1010
24 h	0.3209	0.5479	—	—	—	—
36 h	0.0140	0.4209	—	—	—	—
48 h	—	0.1602	—	—	—	—
Total	11.7612	17.0930	5.9096	7.6005	7.1787	7.8754

Note: “—” means not detected.

### *In Vivo* Metabolites of Anwulignan by Metabolomic Method

**FIGURE 5:**
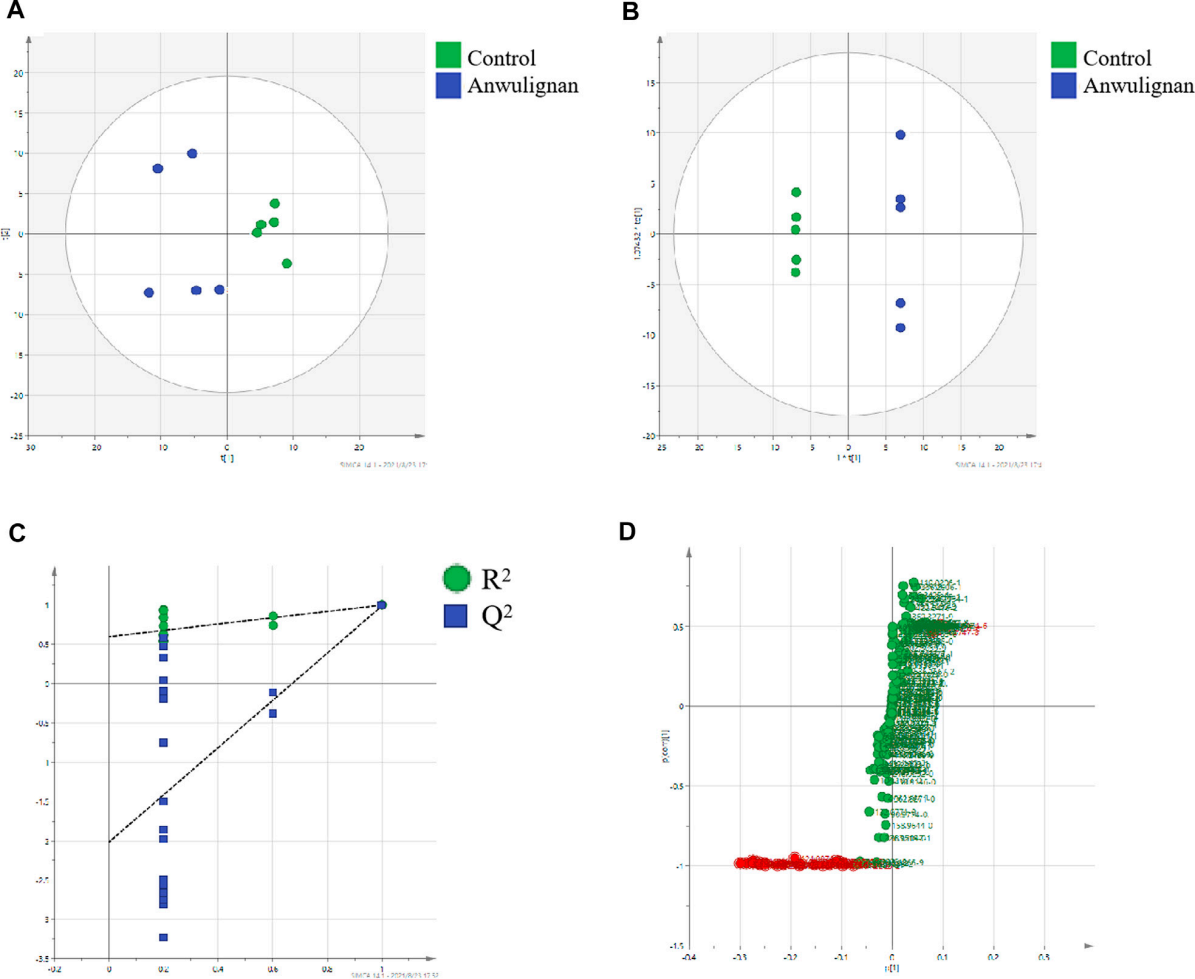
Multivariate statistical analysis control and anwulignan group: **(A)** PCA score plot; **(B)** OPLS-DA score plot; **(C)** cross-validation plot of OPLS-DA model; and **(D)** S-plot of OPLS-DA.

In order to find the metabolites of anwulignan in mice after the intragastrical administration, a metabolomic method based on UHPLC-Q-Orbitrap-MS/MS was used to analyze the samples, and the multivariate statistical analysis of anwulignan was carried out by metabonomic methods, as shown in Figure 5.

Taking the liver samples as an example, unsupervised PCA method was used for the matrix analysis on the data of liver samples from control mice and those treated with anwulignan at 4 h after the intragastrical administration to analyze the relationship among the data, and it could be understood from the score chart of PCA that the samples were well separated between the two groups (Figure 5A). Because PCA could not be used for the analysis of the unknown grouping samples, an OPLS-DA method was used to analyze the data in the two groups to obtain the score chart of OPLS-DA (Figure 5B), which indicated that there were significant differences between the control group and the anwulignan group, and the validity of the model was evaluated by the external model verification method (arrangement of the experiment). As shown in Figure 5C, both slopes of the two regression lines were larger; R^2^ and Q^2^ produced by the left-end alignment were smaller than those by the right-end alignment, and the difference between the two values at the right-most end was smaller, indicating that the model was valid. In order to determine the metabolites of anwulignan in the liver to the greatest extent, S-plot diagram was used for the analysis (Figure 5D). In the S-plot diagram, each point represents an ion, and the farther away from the zero point, the greater the contribution of ions to the fractionation. The ions existing in the liver of mice after the intragastric administration of anwuzhisu, not in the liver of mice in the blank group, were taken as the potential metabolites, and the information on their precise molecular weight and MS/MS fragmentation ions was analyzed.

**FIGURE 6:**
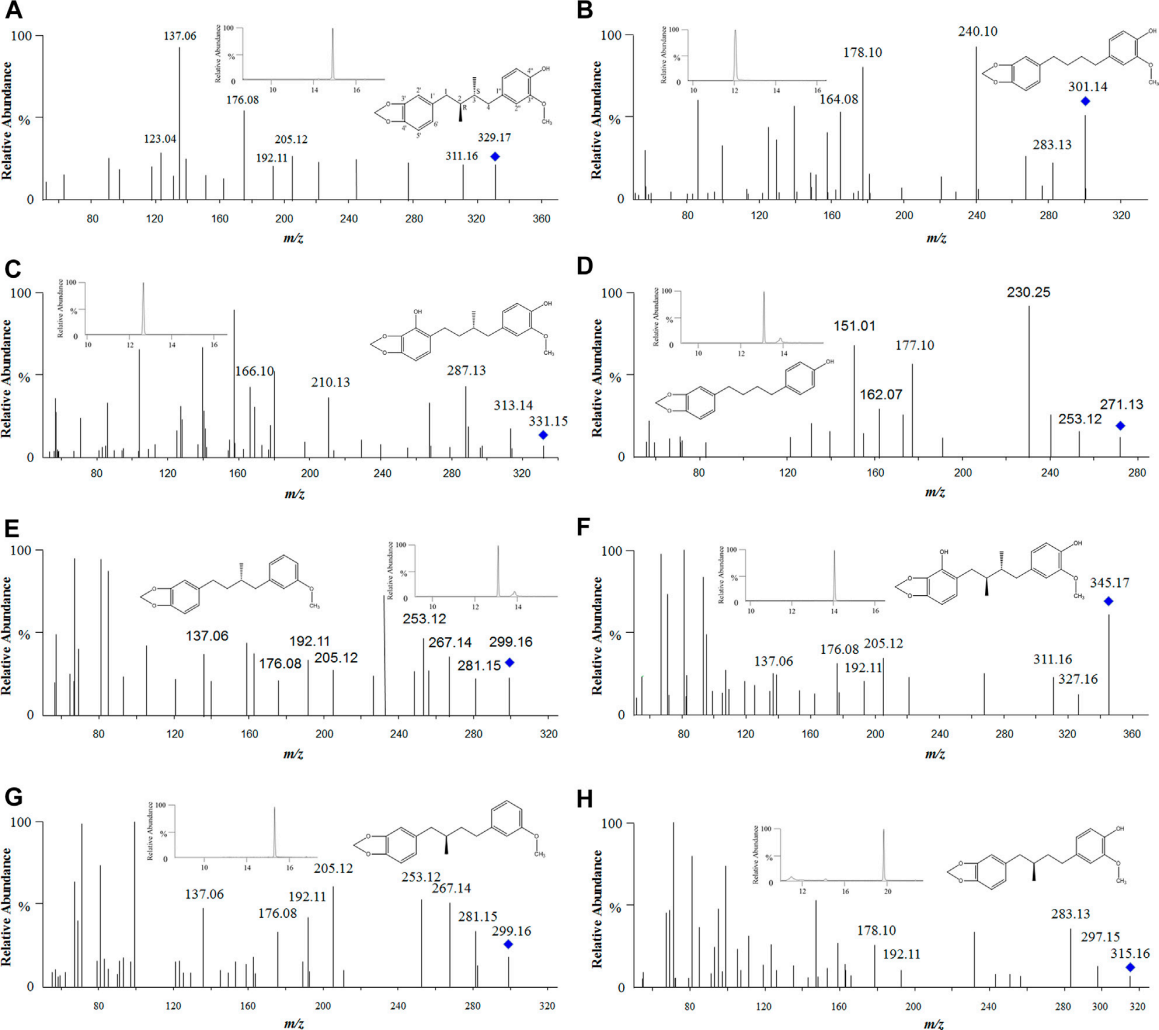
Tandem mass spectra of anwulignan and its metabolites. Note: **(A)** anwulignan at *m/z* 329; **(B)** M1 at *m/z* 301; **(C)** M2 at *m/z* 331; **(D)** M3 at *m/z* 271; **(E)** M4 at *m/z* 299; **(F)** M5 at *m/z* 345; **(G)** M6 at *m/z* 299; and **(H)** M7 at *m/z* 315.

**FIGURE 7:**
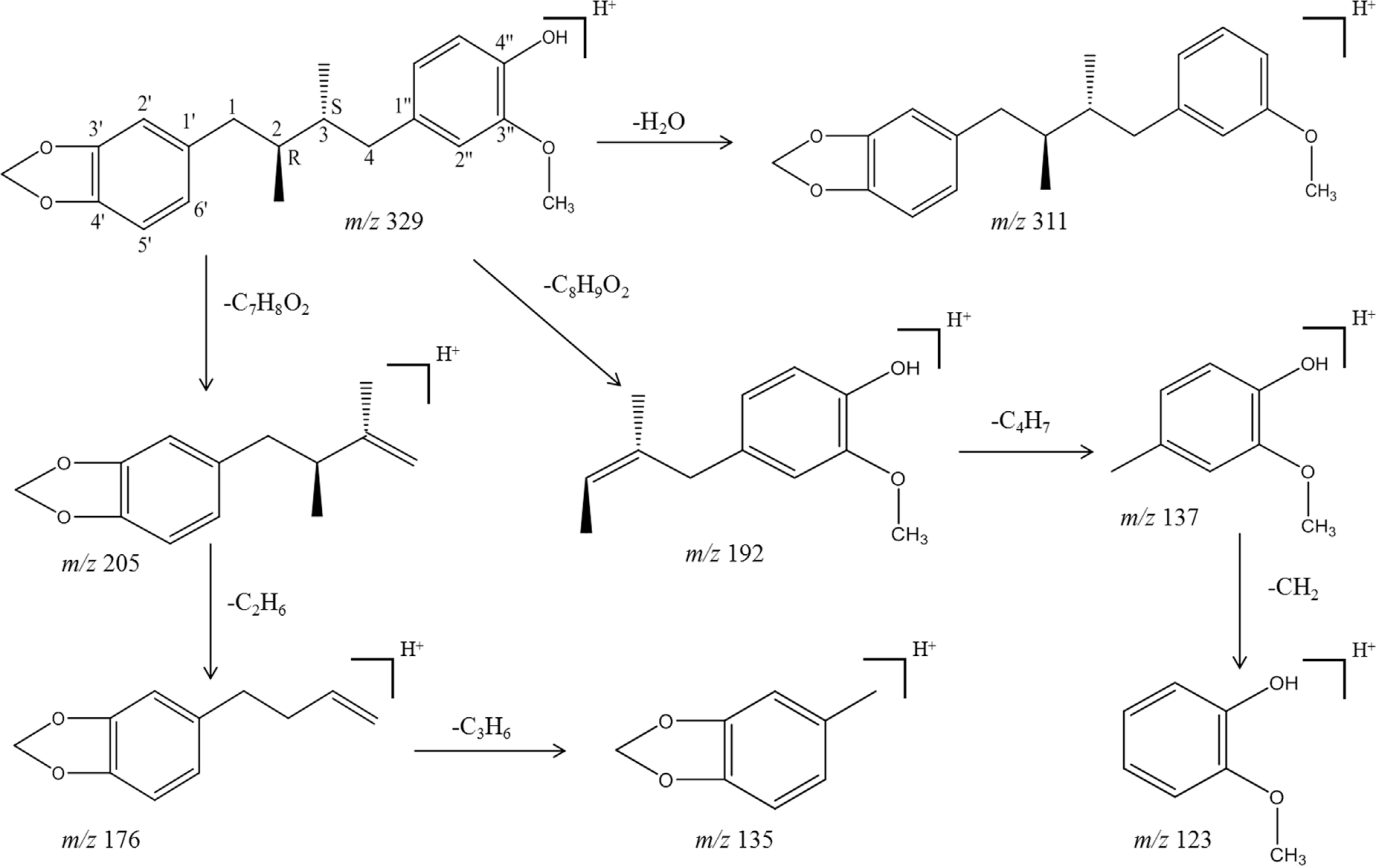
Proposed mainly MS/MS fragmentation pathways for anwulignan.

As shown in Figure 6A, anwulignan presented as [M + H]^+^ in positive-ion mode and its mass to charge ratio (*m/z*) was 329, and fragment *m/z* 311 might be generated from *m/z* 329 losing H_2_O (18 Da) at C-4″ position, *m/z* 205 from *m/z* 329 losing C_7_H_8_O_2_ (124 Da) at C-4, *m/z* 176 from *m/z* 205 losing C_2_H_5_ (29 Da) at C-2 and C-3, *m/z* 135 from *m/z* 176 losing C_3_H_5_ (41 Da) at C-1, *m/z* 192 from *m/z* 329 losing C_8_H_9_O_2_ (137 Da) at C-2, *m/z* 137 from *m/z* 192 losing C_4_H_7_ (55 Da) at C-4, and *m/z* 123 from *m/z* 137 losing CH_2_ (14 Da) at C-1″. The fragmentation mechanism of anwulignan is simulated and shown in Figure 7.

A total of seven metabolites of anwulignan were detected in the blood and liver samples, and the relevant information on these metabolites is shown in Table 6.

**TABLE 6:** Characterization of anwulignan and identified metabolites by UHPLC-Q-Orbitrap-MS.

Lignans	Rt (min)	Formula	*m/z* [M + H]^+^	Error (ppm)	Detection time
Anwulignan	11.91	C_20_H_24_O_4_	329.17	3.6	0.5–48 h (plasma, liver, and feces)
M1	12.09	C_18_H_20_O_4_	301.14	2.1	2 h (liver)
M2	12.69	C_19_H_22_O_5_	331.15	−1.5	1 h (plasma, liver)
M3	12.96	C_17_H_18_O_3_	271.13	3.4	4 h (liver), 4–8 h (feces)
M4	13.07	C_19_H_22_O_3_	299.16	−2.8	4 h (liver)
M5	14.09	C_20_H_24_O_5_	345.17	0.9	4 h (liver)
M6	15.25	C_19_H_22_O_3_	299.16	1.6	4 h (liver), 4–8 h (feces)
M7	19.11	C_19_H_22_O_4_	315.16	−2.2	4 h (liver)

The elution time of M1 (*m/z* 301) was 12.09 min (Figure 6B), suggesting that M1 should be a metabolite of anwulignan of losing two methyl groups, and fragment ion at *m/z* 283 might be generated from that at *m/z* 301 of losing H_2_O (18 Da) at C-4″, *m/z* 178 from *m/z* 283 of losing C_7_H_5_O (105 Da) at C-4, and *m/z* 164 from *m/z* 178 of losing a methyl group (14 Da).

The elution time of M2 (*m/z* 331) was 12.69 min (Figure 6C), suggesting that M2 should be a demethylation metabolite of anwulignan after hydroxylation, and fragment ion at *m/z* 313 might be generated from that at *m/z* 331 of losing H_2_O (18 Da) at C-4″, *m/z* 287 from *m/z* 331 of losing CH_2_ and OCH_2_ (44 Da), and *m/z* 210 from *m/z* 331 of losing C_7_H_5_O_2_ (121 Da) at C-1.

The elution time of M3 (*m/z* 271) was 12.96 min (Figure 6D), suggesting that M3 should be the metabolite of anwulignan of losing a methoxyl group at C-3″, and a methyl group at C-2 and C-3, respectively, and fragment ion at *m/z* 253 might be generated from that at *m/z* 271 of losing a H_2_O (18 Da) at C-4″, *m/z* 177 from *m/z* 253 of losing C_6_H_4_ (76 Da) at C-4, and *m/z* 162 from *m/z* 253 of losing C_7_H_7_ (91 Da) at C-3.

The elution time of M4 (*m/z* 299) was 13.07 min (Figure 6E), suggesting that M4 should be a dehydroxylation and demethylation metabolite of anwulignan, and fragment ion at *m/z* 281 might be generated from that at *m/z* 299 of losing H_2_O (18 Da) at C-4″, fragment ions at *m/z* 267 and 253 might be generated from those at *m/z* 281 of losing one and two methyl groups, respectively, and the fragmentation characteristics of fragment ions at *m/z* 205, 192, 176, and 137 might be basically consistent with those of anwulignan. The elution time of M6 (*m/z* 299) was 15.25 min (Figure 6G), and the fragmentation characteristics of fragment ions at *m/z* 281, 267, 253, 205, 192, 176, and 137 were consistent with those of M4, but the retention time was different from that of M4, so M6 might be the isomer of M4.

The elution time of M5 (*m/z* 345) was 14.09 min (Figure 6F), indicating that M5 should be a hydroxylation metabolite of anwulignan, fragment ion at *m/z* 327 might be generated from that at *m/z* 345 of losing H_2_O (18 Da) at C-4″, and the fragmentation characteristics of the other fragment ions at *m/z* 311, 205, 192, 176, and 137 were consistent with those of anwulignan.

The elution time of M7 (*m/z* 315) was 19.11 min (Figure 6H), indicating that M7 should be a metabolite of anwulignan of losing a methyl group, and fragment ion at *m/z* 297 might be generated from that at *m/z* 315 of losing H_2_O (18 Da) at C-4″, *m/z* 283 from *m/z* 297 of losing a methyl group (14 Da), *m/z* 192 from *m/z* 315 (123 Da) of losing C_7_H_7_O_2_ at C-4, and *m/z* 178 from *m/z* 192 of losing a methyl group (14 Da).

**FIGURE 8:**
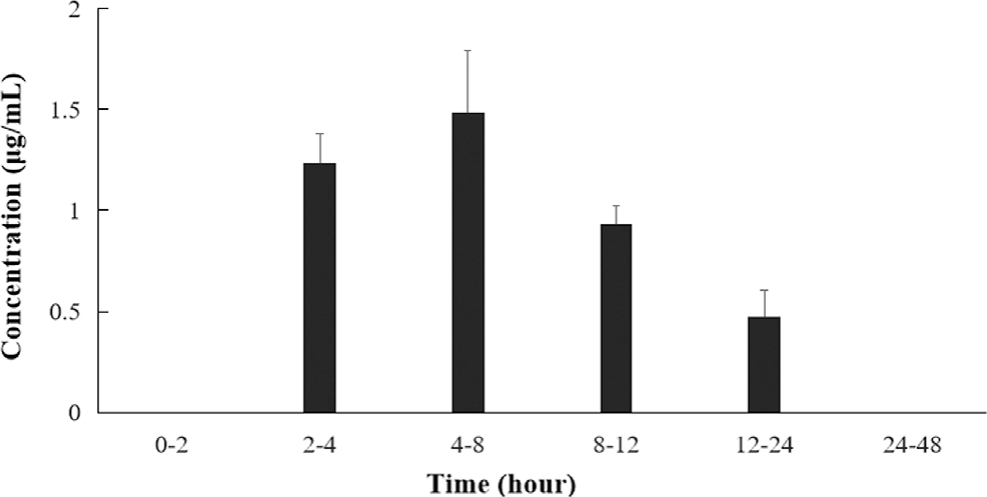
Anwulignan concentrations in the feces of mice after the intragastric administration.

The excreta of mice within 48 h after the intragastric administration of anwulignan were taken for analysis by HPLC. The results showed that anwulignan prototype and its metabolites were not found in the urine of mice within 48 h, and anwulignan prototypes, M3 and M6, were found in the feces, as shown in Figure 8.

## Discussion

The results on the pharmacokinetics of anwulignan in mice indicated that UHPLC-Q-Orbitrap-MS/MS could be well suited for this study. Non-compartment model was used to study the pharmacokinetic characteristics of anwulignan in mice, and it was found that anwulignan was absorbed slowly in mice (T_max_ = 3 h), its duration was long (*t*_1/2_ = 7.1 h), and the prototype of anwulignan could still be detected in the blood until 24 h, indicating that the elimination rate of anwulignan should be relatively slow and there may be an enterohepatic circulation involved in it. Based on the fact that the content of anwulignan in the liver was higher than that in the other tissues, and its residence time was longer, it could be speculated that there may be a hepatointestinal circulation in the metabolism of anwulignan in the body of mice, so it has a long half-life. Its AUC_0−t_ and AUC_0−∞_ were 9.37 ± 2.36 and 12. 58 ± 3.51 mg/Lh, respectively, suggesting that more anwulignan can enter the systemic circulation. Apparent distribution volume (V_d_) can be used to infer the extent of drug distribution in the body, and the V_d_ value is close to 0.8–1.0 L/kg if a drug is evenly distributed in the body and the concentration of a drug in tissues is higher than that in blood when the V_d_ value is greater than 1.0 L/kg, which may indicate that the drug is widely distributed in the body or the tissue protein is highly bound to the drug (Weiss et al., 2021). In this study, the V_d_ of anwulignan was 32.81 ± 4.79 L/kg, indicating that anwulignan has strong lipophilicity, so it is easy for it to enter cells and be widely distributed in tissues through blood circulation. The relative bioavailability of anwulignan was 23.8%, which may be due to the incomplete absorption in the gastrointestinal tract or the obvious first-pass effect on the liver. This result is also reflected by the distribution data of anwulignan in the main organs, which is consistent with that reported by Song et al. (2020).

After the intragastric administration of anwulignan, the prototype of anwulignan could be detected in all major organs of mice, indicating that anwulignan is widely distributed. The distribution of a drug in tissues is related to the blood circulation, vascular permeability, and drug protein binding (Tang and Cao, 2021), and the differential distribution of compounds in various organs mainly depends on the blood flow rate and the concentration of compounds in the organs and the affinity of compounds to the organs. Liver, kidney, and brain are organs with a rapid blood circulation, so anwulignan could be detected quickly in these tissues after the intragastric administration. The order of the concentration of anwulignan in the organs from high to low was liver > heart > brain > kidney > lung > spleen, and the concentration in liver was the highest, indicating that anwulignan may experience a slow elimination in the liver. This result may explain the reason why anwulignan has a therapeutic effect on liver injuries. The liver may be an accumulation site of anwulignan, so taking a large dosage of anwulignan may induce some adverse reactions related to liver metabolism. The content of anwulignan in the spleen was lower, indicating that the tissue distribution of anwulignan after the intragastric administration is targeted. It is worth noting that the concentration of anwulignan in the brain tissue is higher than that of other *Schisandra* lignans in the brain tissue (Lu et al., 2013; Liu et al., 2019; Liang et al., 2021), suggesting that anwulignan has a strong ability to penetrate the blood–brain barrier to play a pharmacological role in the central nervous system, which may provide a reference for the future study on the efficacy of anwulignan.

The study on the excreta of mice showed that anwulignan could be not detected in the urine of mice, but could be detected in the feces, which may be the result of the poor water solubility of anwulignan. It was found in this study that the concentration of anwulignan was highest in feces samples during 4–8 h after the intragastric administration, and anwulignan could still be detected during 12–24 h, further confirming that anwulignan may undergo an obvious enterohepatic circulation, combined with the previous results of this study. The total amount of anwulignan excreted from the body in the form of prototype drug was far less than the dosage, indicating that its excretion from the body is mainly through a metabolic elimination.

**FIGURE 9:**
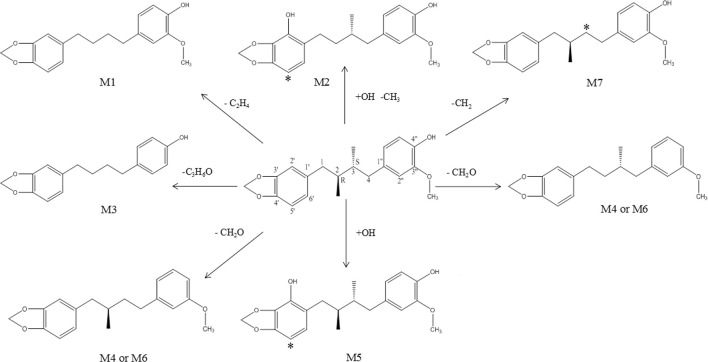
Proposed metabolic pathway of anwulignan in mice. Note: * the potential metabolic site.

Seven metabolites of anwulignan were found in this study, of which only M2 was detected in the blood of mice and the other metabolites were found in the liver, suggesting that the main component of anwulignan to play a pharmacological role should be its prototype or M2 (except the liver), and the effect of the other metabolites should mainly act on the liver or act on the intestinal flora through the way of enterohepatic circulation, so as to indirectly exert its effect on other tissues and organs. Combined with the results of *in vivo* distribution experiment, anwulignan was primarily distributed in the liver after entering the body, and its metabolites were also primarily distributed in the liver, indicating that the liver may be the main organ in which anwulignan can play a role. Some studies have found that anwulignan has an obvious inhibitory effect on carboxylesterases 2 in the liver, and the inhibitory effect is the strongest among many *Schisandra* lignans (Fu et al., 2019). Anwulignan is mainly metabolized in the way of demethylation, hydroxylation, dehydroxylation, and demethoxylation, and studies on other *Schisandra* lignans have also shown that demethylation and hydroxylation are the main metabolic ways of lignans (Liu et al., 2014; Su et al., 2016; Liu et al., 2019; Wang et al., 2020). It was the first time that the metabolites of anwulignan in the body of animals were studied, and the main metabolic pathways are shown in Figure 9.

## Conclusion

UHPLC-Q-Orbitrap-MS/MS was employed to investigate the pharmacokinetics and tissue distribution of anwulignan in mice. It was found that anwulignan was slowly absorbed in mice and widely distributed in various tissues and organs, especially in the liver. The main metabolites of anwulignan *in vivo* were analyzed for the first time, and seven metabolites were found. The results indicate that demethylation, hydroxylation, dehydroxylation, and demethoxylation may be the main metabolic ways of anwulignan, and the majority of its prototypes and metabolites may be excreted through feces.

## Data Availability

The original contributions presented in the study are included in the article/Supplementary Material; further inquiries can be directed to the corresponding author.
